# Student Perceptions of the School eHealth Education Program Pakistan (eSHEPP): A Qualitative Study of Acceptability, Feasibility, and Awareness of Non-Communicable Diseases

**DOI:** 10.21203/rs.3.rs-9707948/v1

**Published:** 2026-06-05

**Authors:** Muhammad Shahid Khan, Aysha Almas, Zainab Samad, Kanecia Obie Zimmerman, Tazeen Saeed Ali

**Affiliations:** Aga Khan University; Aga Khan University; Aga Khan University; Duke University; Aga Khan University

**Keywords:** Adolescent health, school-based intervention, digital health education, health promotion, Pakistan

## Abstract

**Background:**

Noncommunicable diseases are a leading cause of premature morbidity and mortality, and many associated risk behaviors emerge during adolescence. In Pakistan, school health education remains limited and largely lecture-based, leaving adolescents insufficiently equipped to adopt healthy behaviors. To address this gap, the School eHealth Education Program Pakistan, a multimedia and mobile application–supported intervention, was developed. This study explored secondary and higher secondary students’ perceptions of the program’s acceptability, feasibility, and perceived usefulness in improving awareness of noncommunicable diseases following implementation in school settings.

**Methods:**

A qualitative explanatory study was conducted in four schools in Karachi (two secondary and two higher secondary; two all-girls and two all-boys schools). The program was delivered over eight weeks through six classroom sessions. Each 20–30-minute session included a short dramatized video, followed by guided discussion and interactive quizzes facilitated by a trained facilitator and teacher representative. Twenty-seven students were purposively sampled to ensure variation by school type and gender. Data were collected through four focus group discussions conducted after the intervention using semi-structured guides in Urdu. Thematic analysis was performed using NVivo through a hybrid deductive–inductive approach.

**Results:**

Students described the program as acceptable and easy to understand, highlighting the engaging dramatized videos, clear language, and supportive subtitles. Participants reported increased awareness of noncommunicable diseases and related risk factors. Some students expressed intentions to make healthier dietary choices, increase physical activity, and discourage smoking among peers. The program was considered feasible within existing school schedules and required minimal technological or personnel resources. Students suggested expanding health topics, slightly extending session duration, and incorporating additional interactive features to enhance engagement.

**Conclusions:**

The program showed strong acceptability, feasibility, and perceived educational value in under-resourced school settings. With improvements in interactivity and integration into school curricula supported by teachers and parents, it may provide a promising model for adolescent health education in similar contexts.

## Introduction

Adolescents constitute a rapidly expanding population in low- and middle-income countries (LMICs), and their health behaviors are likely to shape the trajectory of the noncommunicable disease (NCD) epidemic[1, 2]. NCDs account for the majority of global deaths, with LMICs disproportionately affected by premature mortality [3–5]. A substantial proportion of premature adult deaths can be traced to risk behaviors established during childhood and adolescence including physical inactivity, unhealthy diet, tobacco and substance use, and overweight or obesity [6, 7]. The World Health Organization (WHO) has identified the rising prevalence of NCD risk factors among adolescents as a critical global health concern [8]. Adolescence represents a window of opportunity for establishing lifelong healthy behaviors, as young people navigate complex physical, psychological, and social transitions [9]. WHO estimates indicate that NCDs cause 74% of global deaths, with approximately 82% of premature NCD deaths occurring in LMICs, underscoring adolescence as a formative period for future health trajectories [1, 6, 10].

Health education is widely recognized as a central strategy for the prevention of noncommunicable diseases, particularly when delivered during adolescence, a period when many health behaviors are established [1]. Evidence suggests that adolescents require both knowledge and practical skills to make informed health decisions, with interactive, school-centered programs consistently outperforming traditional didactic approaches [11]. Strategies such as model-driven trials and participatory techniques are essential for enhancing both short- and long-term health outcomes [12]. In line with WHO’s Health-Promoting Schools framework, well-designed, student-centered programs can foster healthier behaviors; however, many education systems in LMICs remain under-resourced and inconsistently implemented [13, 14].

In Pakistan, structured school health programs are scarce, and evidence on effective delivery strategies is limited. National analyses have described school health as an “ignored domain”, emphasizing the need for scalable, context-sensitive interventions that address both knowledge and program feasibility [15]. Digital technologies offer innovative, accessible, and cost-effective solutions to enhance student health literacy. They can promote healthy eating, physical activity, and chronic disease prevention, while leveraging mobile connectivity, social media, and gamified learning to engage adolescents [16, 17]. Evidence shows that digital and mass media campaigns can positively shape health norms and behaviors [1, 18], and adolescents are increasingly accessing mobile apps, peer networks, text-based health programs, and online mental health resources [19–21]. eHealth interventions have demonstrated potential to improve adolescent health outcomes and reduce health inequities, particularly in LMICs such as Pakistan [1, 16, 20–25]. This opportunity is significant in Pakistan, where nearly one in five people is aged 10–19 years, and mobile connectivity is widespread (≈ 198 million cellular subscribers and > 134 million mobile broadband users as of June 2025). However, digital divides persist, especially by gender and digital literacy [26–28]. Systematic reviews highlight that well-designed eHealth and mHealth interventions can enhance physical activity, mental health, and self-management of NCDs when usability and engagement are prioritized [29–31].

Despite growing recognition of adolescent health needs, school-based health education in Pakistan remains fragmented and underdeveloped, often lacking interactive or participatory methods [32]. Health topics are rarely integrated into core curricula, and public-sector schools face constraints including limited infrastructure, trained staff, and cultural sensitivities around health discussions [33]. School curricula are typically exam-oriented, leaving limited space for health education, and past interventions have struggled with uptake, sustainability, and contextual relevance. Consequently, students remain underprepared to make informed health choices. Early evidence suggests that digital, school-based lifestyle programs are feasible and scalable, though acceptability and contextual fit remain key determinants of success [34].

With the rapid rise in mobile phone usage across Pakistan [35], digital health platforms present an opportunity to deliver scalable, engaging, and culturally relevant health education to adolescents [16, 17]. However, evidence remained limited regarding students’ perceptions, acceptability, and feasibility of integrating digital health education within school systems in low-resource settings. To address this gap, we developed the School eHealth Education Program Pakistan (eSHEPP) in 2024. The program was created by a multidisciplinary team of public health researchers, educators, and digital health specialists based in Karachi, in collaboration with local educational authorities, teachers, parents, and students [36]. eSHEPP is part of a broader mixed-methods research program designed to develop, pilot, and evaluate a culturally appropriate digital school-based intervention to improve adolescents’ awareness of noncommunicable disease risk factors and healthy lifestyle behaviors. The program was developed in response to the scarcity of structured school health programs in Pakistan and the need for scalable, context-sensitive approaches to adolescent health education. Development followed a participatory approach, involving focus groups and interviews with students, teachers, parents, and school administrators to ensure contextual relevance, feasibility, and acceptability [37, 38].

eSHEPP comprised six multimedia classroom sessions delivered over eight weeks through an interactive digital platform integrating narrative videos, quizzes, animations, and facilitator-led discussions. Content addressed healthy diet, physical activity, avoidance of tobacco and substance use, and overall well-being, presented through culturally relevant Urdu narratives with optional English subtitles. Preliminary exploratory work identified infrastructure constraints, implementation barriers, and facilitators such as student familiarity with digital technology and positive stakeholder support, which informed refinements including offline access, bilingual content, and additional interactive features [37]. This study aimed to explore students’ perceptions of the usefulness, acceptability, and feasibility of the program following delivery in school settings. The study was guided by the Technology Acceptance Model (TAM) and the Task–Technology Fit (TTF) framework. TAM explains how perceived usefulness and ease of use influence technology adoption, while TTF evaluates whether a technological tool aligns with user tasks and needs [39, 40]. Although these models are widely applied in digital health research, they primarily focus on technology adoption rather than broader behavioral determinants, and their application in adolescent health education requires careful contextual interpretation. By exploring students’ perceptions, this study addressed the evidence gap on digital school-based health education in Pakistan and informed future implementation strategies in low-resource settings.

## Methods

### Study design

This study employed a qualitative explanatory design with a descriptive approach, which is well-suited to examining participants’ perspectives and lived experiences. The explanatory component provided deeper understanding of how and why students interacted with eSHEPP’s digital content, app features, and health messages.

### Study Setting

The study was conducted in four public (government) schools in Karachi, Pakistan’s largest city. These schools serve middle- to lower-middle-income communities and, like most Pakistani public schools, operate with limited resources and minimal access to digital learning tools. Prior to eSHEPP, health education was limited to occasional didactic lectures. This resource-constrained context makes public schools an important setting for evaluating digital health interventions.

### Theoretical framework

**Figure 1:**
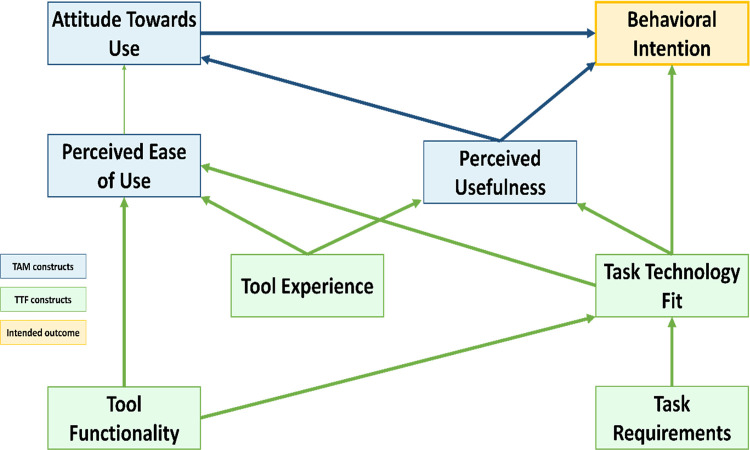
Integrated model of the Technology Acceptance Model (TAM) and Task–Technology Fit (TTF), adapted from Shih and Chen (2013)

The study was guided by an integrated framework combining the Technology Acceptance Model (TAM) and the Task–Technology Fit (TTF) model (Fig. 1) adapted from Shih and Chen (2013) [40]. TAM constructs (perceived usefulness, perceived ease of use, and intention to use) informed questions about students’ views on the relevance and usability of eSHEPP, while TTF constructs (alignment with learning needs, task–technology fit, and perceived impact on performance) shaped questions about whether the program supported students in applying health knowledge.

The integrated model posits that perceived usefulness and ease of use influence task–technology fit, which in turn affects engagement and learning performance. This framework also guided a hybrid coding strategy, combining deductive codes derived from TAM–TTF constructs with inductive themes emerging from student narratives. A detailed codebook, including all themes, subthemes, definitions, and mapped TAM-TTF constructs, is provided in Supplementary Material 5.

While TAM and TTF provide useful frameworks, they were originally developed in organizational contexts and may not fully capture peer influences or developmental dynamics shaping adolescent engagement with digital tools. These factors were considered during analysis.

### Sampling technique and Schools Selection

The study was conducted in the four schools where the eSHEPP intervention was delivered: two secondary schools and two higher secondary schools (one all-boys and one all-girls per level). These districts were included after formal approval from the Directorate of School Education (DSE), Karachi, which authorized and directed program implementation.

The research team was not involved in school selection, which was determined administratively by the DSE and respective District Education Offices (DEOs). As detailed in the project protocol [36], a total of eight schools were randomized into intervention (n = 4) and control (n = 4) groups, and this qualitative study was conducted only in the four intervention schools to explore acceptability, feasibility, and usefulness in real-world implementation.

### Participants and Sampling

A total of 27 students participated (12 males, 15 females), purposively selected to capture diverse perspectives. Approximately 8 students were included per focus group, consistent with recommendations to balance rich discussion and manageability [41]. All eligible students were invited meeting inclusion criteria: enrollment in secondary or higher secondary grades, active participation in all six eSHEPP sessions, and provision of parental consent and student assent. Students were excluded if they missed sessions or could not provide consent. Of 32 eligible students approached, 27 consented and attended (response rate 84.4%); three did not provide consent, and two were absent on the day of the discussions. Participants included students with varying levels of engagement and attitudes toward the program. Supplementary Material 1 summarizes participant characteristics by focus group and gender. Students received USB drives as appreciation; no financial or transport compensation was provided.

### Intervention delivery

The eSHEPP is a digital, school-based health education intervention designed to improve awareness of NCDs among adolescents and promote healthy behaviors. The intervention was implemented according to a previously published mixed-methods protocol [36]. Development of the intervention was informed by an earlier qualitative exploration phase, which identified barriers and facilitators to implementing digital health education in secondary and higher secondary schools in Karachi and examined stakeholders’ views on the content and design of the proposed eSHEPP program [37]. These insights guided refinement of educational videos, session structure, and the digital platform.

The program was delivered through a custom-built eHealth application accessible on mobile devices and web browsers. The app hosted Urdu-language educational videos, interactive quizzes, and pre- and post-assessments, enabling students to review content and monitor progress. It was hosted on a secure university-managed server with restricted access.

The curriculum comprised six structured multimedia sessions delivered over eight weeks during regular classroom hours. Each 20–30 minute session included: a brief educational video, a facilitated discussion, and an interactive quizzes co-led by a trained facilitator and a teacher representative. Videos used dramatized storytelling and animation to enhance engagement, featuring an adolescent protagonist who encouraged his family to adopt healthier habits (diet, physical activity, avoiding smoking/substance use). Core topics included NCD awareness, healthy eating (Eat Smart), physical activity (Keep Moving), substance avoidance (Run Away from Smoking and Drugs), and overall wellness (Stay Well).

After sessions, students accessed the app individually to review materials, complete, and reinforce learning. Facilitators received standardized training, recorded attendance, followed up with absentees, and completed post-session fidelity checklists to ensure consistent delivery across schools.

Feasibility and potential efficacy were previously evaluated in a pilot cluster randomized controlled trial; full results will be reported separately. The current qualitative study explores experiences, acceptability, and engagement of students with eSHEPP using standardized procedures.

### Data collection

A semi-structured focus group discussion (FGD) guide was developed based on TAM and TTF constructs and aligned with eSHEPP’s digital content. It was translated into Urdu and piloted with four students from a non-participating school to assess clarity, cultural relevance, and contextual fit; only minor language adjustments were made. The complete guide is provided in Supplementary Material 6.

FGDs were conducted 3–4 weeks after the final eSHEPP session in February 2025 to explore students’ views on usefulness, acceptability, feasibility, and usability of the mobile and web versions, including engagement with videos, storyline interpretation, and application to daily life. Each FGD lasted 30–45 minutes in a private room on school premises during regular hours, with no teachers or administrators present.

FGDs were moderated by an external trained qualitative researcher (MSK) and assisted by a female note taker who co-facilitated when possible, particularly in all-female groups. Gender-matched facilitation was achieved for all female groups; in one all-male group full matching was not possible due to facilitator availability, as documented in reflexive notes. Potential researcher influence was mitigated through external facilitation, gender-sensitive procedures, and private settings. Written parental consent and student assent were obtained, and all sessions were audio-recorded with permission.

### Data Processing and Saturation

Audio recordings were transcribed verbatim in Urdu within two weeks by a bilingual transcriptionist and translated into English by a separate bilingual researcher. A second team member cross-checked all transcripts against the recordings, and discrepancies were resolved through discussion. Data collection continued until thematic saturation, defined as the point when no new codes, subthemes, or concepts emerged. Saturation was assessed iteratively: after each FGD, the moderator and note taker documented emerging patterns, and weekly meetings with co-investigators (TSA, AA) compared new codes to the developing codebook. Minimal new codes emerged after the third FGD; the fourth confirmed saturation.

### Data analysis

A hybrid deductive–inductive thematic analysis was conducted following Braun and Clarke’s six-phase framework [42]. Deductive codes were derived from TAM–TTF constructs, and inductive codes emerged from student narratives. Analysis phases included familiarization (repeated reading), generating initial codes (two researchers independently coded the first transcript), searching for themes (grouping codes), reviewing themes (checking against coded extracts and full dataset), defining/naming themes, and producing the report with illustrative quotations.

NVivo (version 10) was used to manage transcripts. Three consensus meetings with the lead researcher (MSK) and co-investigators (AA, TSA) resolved coding discrepancies. Feasibility was assessed via perceived acceptability and usability, not objective metrics. Quotations included participant age and FGD identifier to provide context while maintaining anonymity. The analysis adhered to COREQ guidelines (Supplementary Material 4).

### Trustworthiness and rigor

To maintain rigor, the study followed Lincoln and Guba’s four criteria for establishing trustworthiness in qualitative research [43]. Credibility was enhanced through prolonged engagement, gender-matched facilitation, team debriefings, and member checking, with minor refinements made based on participant feedback. Dependability was supported by detailed documentation of sampling, data collection, and analysis, maintained in NVivo and research logs. Confirmability was ensured via reflexive journaling by the lead researcher and regular peer debriefings. Transferability was promoted through thick description of study context, participants, and intervention implementation.

### Positionality and Reflexivity

The lead researcher (MSK), an external male public health professional with experience in adolescent digital interventions, served as an outsider, minimizing pre-existing biases while building rapport with participants. The female note taker provided additional outsider perspective, particularly during all-female groups. In one all-male group, full gender matching was not possible, documented in reflexive notes. Reflexive journaling captured field notes, personal reactions, assumptions about adolescent digital literacy, and analytical decisions. Team debriefings with co-investigators (TSA, AA) addressed how researcher positionality might influence interpretation, ensuring findings were grounded in participants’ perspectives and enhancing confirmability.

### Ethical considerations

Ethical approval was obtained from the Aga Khan University Ethical Review Committee (Ref No. 2023-9277-27367). Written informed consent from parents and assent from students were obtained. USB drives were provided as appreciation; no financial or transport compensation was given. Data were stored on password-protected systems, and audio recordings were destroyed following transcription and verification. Any modifications to the protocol required approval from the project steering committee and ethics committee.

## Results

After two months of delivering eSHEPP in four schools (two secondary and two higher secondary; two all-girls and two all-boys), 27 students who attended all six sessions participated in four FGDs. The FGDs included two male groups (n = 12) and two female groups (n = 15), capturing gender-specific perspectives. Secondary and higher secondary students were included to explore potential differences across age and school level. Participant demographics are summarized in Supplementary Material 1.

**Figure 2:**
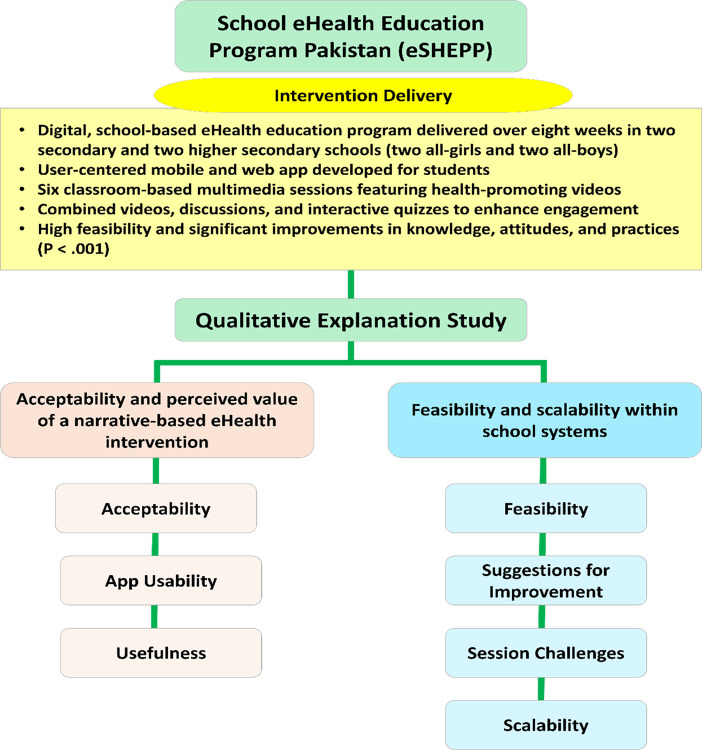
Conceptual framework illustrating intervention delivery and key qualitative themes and subthemes of eSHEPP

Thematic analysis identified two overarching themes encompassing seven interrelated subthemes, as depicted in Fig. 2. The first overarching theme, Acceptability and perceived value of a narrative-based eHealth intervention, comprised the subthemes Acceptability, App Usability, and Usefulness. The second theme, Feasibility and scalability within school systems, included the subthemes Feasibility, Suggestions for Improvement, Session Challenges, and Scalability (Supplementary Material 3). Overall, students reported high acceptability and perceived usefulness, minimal disruption to academic routines, and perceived benefits in knowledge and peer/family influence. Patterns varied slightly by gender and school level: female students highlighted interactive discussions and storytelling as particularly engaging, while older students emphasized self-directed use of the app and comprehension of NCD topics.

### Acceptability and perceived value of a narrative-based eHealth intervention

#### Acceptability

Acceptability captured students’ overall reception and engagement with eSHEPP, reflecting their enjoyment, attention, and interaction with facilitators (TAM: Perceived Usefulness & Ease of Use). Students valued multimedia, interactive learning, and supportive facilitation. Sessions were seen as a refreshing break from traditional classroom routines, especially among female students who reported higher engagement during group discussions.

Students highlighted that visuals, storytelling, and dramatization in videos made health concepts memorable and easy to share at home.

“The information… was very helpful. We shared this with our family members.”

(FGD3, Male)

The novelty of video-based learning sustained attention and motivated discussions beyond the classroom.

“It was something different from regular studies, making it more interesting.”

(FGD1, Female)

Students appreciated facilitators who clarified doubts and encouraged interaction, creating a supportive environment.

“The atmosphere was very friendly.”

(FGD2, Female)

“The program was quite good. It was enjoyable… and it felt good. It refreshed our minds.”

(FGD1, Female)

#### App usability

App usability examines whether the eHealth app interface supported effective learning (TAM: Perceived Ease of Use). Most students, regardless of gender, found the app intuitive, visually clear, and easy to navigate. Older students noted that the simple interface allowed them to focus on content rather than technical issues.

“I would recommend it; the app is very user-friendly and easy to navigate.”

(FGD2, Female)

“The app was straightforward, no confusing steps or complicated features.”

(FGD3, Male)

#### Usefulness

Usefulness measures whether students perceived tangible benefits from the program in terms of knowledge, attitudes, and behavior (TAM: Perceived Usefulness). This subtheme also reflects task–technology fit, as students described how digital content supported their ability to understand and apply health information in daily life (TTF). Behavioral reflections are interpreted cautiously, as perceived changes rather than objectively measured outcomes. All students reported substantial learning gains about NCDs, healthy lifestyles, and mental health, with female participants more frequently noting sharing knowledge at home.

“We learned so much through this program, especially about how smoking, physical activity, and diseases are connected.”

(FGD1, Female)

“Before these sessions, we ate oily foods without realizing they could lead to health problems. Now we understand the risks much better.”

(FGD1, Female)

“We learned it’s important to share our feelings with others; this helps reduce stress and prevents mental health issues.”

(FGD1, Female)

Several participants further described changes in their practices and everyday habits, showing that lessons learned translated into tangible actions at home and with peers.

“After learning about diabetes, I advised my mother to cut back on sugar, and she actually listened!”

(FGD3, Male)

“When I saw my friend smoking, I intervened and invited him to learn more about the risks.”

(FGD4, Male)

### Feasibility and scalability within school systems

#### Feasibility

Feasibility assessed how easily eSHEPP could be integrated into school schedules, considering practical constraints and resources (TTF: Task–Technology Alignment). This reflects strong task–technology fit, highlighting alignment between program design, school timetables, available resources, and students’ learning needs. Students reported that sessions fit smoothly into timetables and required minimal resources. Teacher presence was appreciated, particularly in male schools, for maintaining focus.

“The sessions were like PT (Physical Training) periods, enjoyable but still structured, so they didn’t disrupt our regular studies.”

(FGD2, Female)

“We don’t need expensive equipment or complicated setups, just basics like a laptop and speaker.”

(FGD3, Male)

“When teachers are involved, it makes us feel the program matters - we take it more seriously.”

(FGD2, Female)

Weekly sessions were preferred, balancing retention of knowledge with sustained interest. Videos were clear and concise; dramatization and subtitles improved comprehension. Most students preferred 7–10 minutes, though some suggested slightly longer sessions.

“Weekly sessions help us remember better without losing interest.”

(FGD3, Male)

“The English subtitles were very helpful; they kept us engaged and made it easier to follow along.”

(FGD3, Male)

“A 20-minute session worked well, it’s short enough not to disrupt our schedule.”

(FGD4, Male)

#### Session challenges

Students occasionally reported technical difficulties, background noise, and seating arrangements as barriers. A few students in each FGD reported technical difficulties or background noise, primarily in larger classrooms.

“The poor video and audio quality made it difficult to understand, especially with all the background noise.”

(FGD4, Male)

“When other students talked during sessions, we missed key information.”

(FGD4, Male)

“Older students occupied the front rows, leaving younger students in the back struggling to see the content.”

(FGD4, Male)

#### Suggestions for improvement

Students provided thoughtful recommendations to enhance engagement and effectiveness (TAM-TTF: Refinement of Fit). Female students emphasized interactive storytelling and peer discussion, while male students suggested additional app functionalities and content variety.

“We want more content; currently, it only covers one disease. Expanding to other health issues would be valuable.”

(FGD4, Male)

“We need more stories like Kamran’s journey; one example is good, but more would be better.”

(FGD1, Female)

Some participants also proposed increasing the duration of sessions slightly to allow for deeper discussions and enhanced engagement.

“Slightly longer sessions would make the experience more engaging and enjoyable.”

(FGD2, Female)

Students further emphasized the value of interactive digital features to increase user participation and enjoyment. Suggestions included adding voice recording options, incorporating health-related games, and enabling features for peer interaction and content sharing.

“The app should include a voice recording feature; this would let students explain concepts more clearly.”

(FGD3, Male)

“Adding interactive games to the app would help us learn concepts more effectively.”

(FGD3, Male)

“Adding playback controls like fast-forward or full-speed options would help us review content more efficiently.”

(FGD1, Female)

#### Scalability

Both genders recognized the value of collaborative engagement to ensure program reach and long-term sustainability. Students suggested integrating eSHEPP into the school curriculum and involving teachers and parents to enhance impact.

“Implementing this program in every school would benefit all students equally.”

(FGD4, Male)

“Integrating this program into the school curriculum would give it the importance it deserves, ensuring all students engage seriously.”

(FGD2, Female)

“Teachers can amplify this program’s reach, they already have organized classes where they can share the content.”

(FGD1, Female)

“Parents should receive this too; they’ll share it with the whole family, making the lessons reach more people.”

(FGD3, Male)

**Figure 3:**
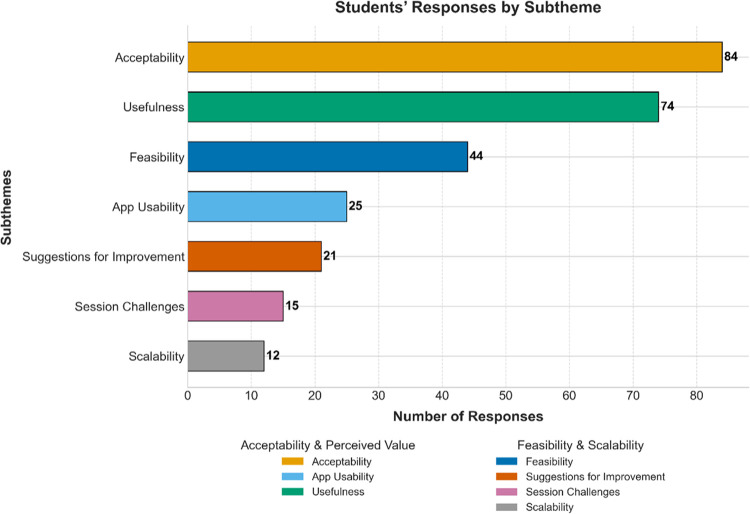
Distribution of students’ responses by subtheme within two overarching themes: Acceptability and perceived value of a narrative-based eHealth intervention and Feasibility and scalability within school systems.

Figure 3 shows how student responses were distributed across subthemes within two main areas: Acceptability and Perceived Value of a Narrative-Based eHealth Intervention and Feasibility and Scalability within School Systems. While our analysis focuses on understanding themes in depth, the frequencies here help illustrate which topics were most prominent in student discussions, without implying any statistical conclusions [42]. Overall, Acceptability and Usefulness were most frequently discussed, indicating strong engagement and perceived benefits, while session challenges were mentioned less often, reflecting occasional constraints. Gender- and school-level differences are noted descriptively without statistical inference. Overall, female students emphasized interactive discussions and storytelling, while older students focused more on self-directed app use and comprehension of NCD topics, suggesting minor variations in engagement and learning preferences by gender and age. Full distribution is provided in Supplementary Material 2.

## Discussion

### Principal Findings

Secondary and higher secondary school students in Karachi responded positively to eSHEPP, describing the intervention as acceptable, feasible, and beneficial. Two overarching themes emerged: high acceptability and perceived value, and strong feasibility with potential for scalability within school systems. Together, these themes reflect the close alignment of the intervention with students’ needs, preferences, and contextual realities. However, these findings reflect perceptions from a small, urban, purposively selected sample and should not be generalized to all Pakistani school settings without further evaluation. Further evaluation in rural, private-sector, and lower-connectivity settings is required before large-scale implementation can be recommended.

Students' positive engagement and perceived ease of use aligned closely with the TAM, with high engagement reflecting perceived usefulness and simple navigation supporting ease of use. The TTF model was also supported, as students emphasized the program’s alignment with school schedules, available infrastructure, and preferred learning styles, facilitating sustained engagement. Perceived behavioral reflections were interpreted cautiously, consistent with the qualitative nature of the study.

The organization of findings into two overarching themes reflected the theory-driven nature of the dataset. Acceptability and usability primarily mapped onto TAM constructs, whereas feasibility and session organization aligned with TTF dimensions. This structure enhanced conceptual clarity and ensured coherence between the analytical framework and empirical findings. Supplementary Material 5 provides a detailed mapping of subthemes to TAM and TTF constructs.

### Comparison With Prior Work

Our findings provided in-depth insights into adolescents’ experiences with eSHEPP, particularly regarding acceptability, engagement, and feasibility. Participants emphasized that interactive content and relatable narratives enhanced motivation and comprehension, consistent with previous digital health interventions [44–48]. Beyond confirming existing evidence, this study extended prior work by demonstrating how narrative-based, Urdu-language digital content can be integrated into public-sector schools in Pakistan with minimal disruption. Unlike some interventions that reported low uptake or sustainability challenges, students in this study highlighted institutional compatibility and teacher support as key facilitators.

For example, adolescents participating in smartphone-based oral health and mental health interventions have highlighted the importance of clear instructions, interactivity, and opportunities for discussion [44–46]. Other qualitative studies have reported that access to culturally relevant and personalized digital tools, along with careful attention to privacy and localization, are key factors influencing adolescents’ motivation and sustained engagement with digital health programs [47, 48]. Students described eSHEPP as different from traditional classes, mainly due to its multimedia content, interactive discussions, and narrative videos. This contrast reflects the lecture-based approach in many Pakistani public schools, where student participation and digital tools are limited. The positive response suggests a need for more interactive, student-centered, and technology-enhanced methods in health education.

The findings also showed that adolescents’ engagement and perceived benefits were shaped by the intervention’s usability, its relevance to the school context, and its practical applicability to daily life. Many students reported influencing their peers and family members, which is consistent with previous qualitative evidence from digital storytelling and mHealth interventions that highlights adolescents as agents of change within their communities [44–48]. Participatory design studies further indicate that aligning digital programs with school schedules, available resources, and local learning contexts enhances both feasibility and scalability [45, 49–51].

While previous studies report improvements in health knowledge and behaviors [52–61], our qualitative findings nuance this evidence by showing that perceived behavior change was mediated by family and peer interactions and reinforced through facilitated discussion. This highlights the importance of blended digital–classroom approaches in LMIC school contexts.

Overall, by integrating qualitative findings with existing quantitative evidence, this work underscored the importance of interactive, contextually relevant, and narrative-driven design in school-based digital health interventions. It also contributed context-specific insights from Pakistani adolescents, extending the existing evidence base and highlighting considerations for implementation in similar settings. Whereas many previous studies focused primarily on individual-level outcomes, our findings emphasize the importance of institutional and social reinforcement.

### Study Contributions and Novelty

To our knowledge, this is the first qualitative study in Pakistan to explore students’ perceptions of a school-based digital health education program using an integrated TAM–TTF framework. By explicitly linking usability, contextual fit, and engagement, this study advances understanding of how theoretical models operate in low-resource school environments. The findings may inform alignment with Pakistan’s National Health Vision (2016–2025) and Digital Pakistan Policy (2018) and to guide the sustainable scale-up of digital adolescent health interventions in schools [62, 63].

The eSHEPP app played a central role in these positive outcomes. As a blended digital learning platform, it combined dramatized storytelling, short educational videos, and interactive quizzes to reinforce key messages. Its Urdu-language interface, simple navigation, and multimedia elements made learning accessible and engaging for students from diverse backgrounds. Beyond delivering information, the app encouraged self-paced review, reflection, and classroom discussion, supporting deeper understanding and retention of health concepts. These design features supported cognitive engagement (TAM) and instructional alignment (TTF), contributing to sustained participation. The findings provide practical guidance for policymakers and program developers seeking culturally responsive digital health interventions.

### Strengths and Limitations

A key strength of eSHEPP is its ability to deliver health education without disrupting academic routines. The program requires only basic equipment and combines app-based learning with in-person facilitation, enhancing its practicality in low-resource school settings. Students also provided valuable suggestions for improvement, including expanding health topics, slightly lengthening sessions, and adding interactive app features such as games, voice recording, or peer chat. These recommendations offer clear opportunities to enhance the program further. Although the study focused specifically on user experience and delivery feasibility, this targeted approach enabled rich, actionable insights that can inform future program adaptation and scaling.

Several limitations should be acknowledged. The study relied on self-reported focus group data, which may be influenced by social desirability and recall bias, and it did not include objective measures of behavior change. Additionally, these feasibility findings are based solely on students’ perspectives and do not represent an institutional or administrative assessment. A full evaluation would require additional information on costs, teacher training, and system-level resources. Facilitator presence, teacher involvement, and the institutional school environment may have shaped students’ responses. Despite efforts to mitigate power dynamics, including the use of external and gender-matched facilitators, institutional settings may have encouraged positive reporting. As with all qualitative studies, findings may also reflect researcher interpretation.

Furthermore, qualitative design captures perceptions but cannot establish causality or generalizable outcomes. The sample comprised only students who completed all six sessions, excluding those who may have disengaged or dropped out. The study relied exclusively on qualitative findings without corresponding quantitative or objective outcome measures, which limit the assessment of actual behavior change or intervention effectiveness. Finally, the urban setting and purposive sampling further restrict generalizability to other school contexts. Future research could strengthen validity by integrating qualitative insights with objective indicators, such as physical activity logs, step counts, or dietary recalls, and by conducting longitudinal and multi-site evaluations, including rural and underserved schools.

### Future Directions

Given that feasibility and scalability were assessed primarily through student perceptions, future research should incorporate operational indicators, such as training costs, infrastructure requirements, and implementation fidelity at scale. Integrating the program into formal curricula, supported by active teacher facilitation and parental reinforcement, may help sustain long-term benefits. This approach aligns with frameworks for sustainable, low-barrier adolescent health promotion [55].

Mixed-method and longitudinal designs are needed to evaluate sustained effectiveness. Aligning eSHEPP with Pakistan’s National School Health Policy and the WHO Global Standards for Health-Promoting Schools could support institutionalization and long-term adoption. Achieving sustainability will require system-level integration and policy support. Evidence from other low- and middle-income countries suggests that embedding digital health education into national curricula, with government and donor backing, can enhance both reach and effectiveness [64]. Ensuring equitable access remains essential, particularly for students without smartphones or optimal classroom environments. Future implementation studies across provinces and school systems are needed before national scale-up can be recommended.

## Conclusion

eSHEPP demonstrated strong acceptability and feasibility among urban public school students in Pakistan, with its narrative-based, multimedia format enhancing engagement and perceived learning. However, these findings represent student perceptions from a small, purposive sample in one city. Future large-scale implementation requires rigorous evaluation using mixed methods and objective outcome measures across diverse settings, including rural schools and varied socioeconomic contexts. With content refinement, increased interactivity, and policy support for curriculum integration, eSHEPP has the potential to serve as a scalable model for adolescent NCD prevention in low-resource school systems.

### Implications for Policy and Practice

School-based digital health education is feasible in low-resource settings, requiring minimal equipment and not disrupting academic routines. Multimedia, app-supported learning effectively engages adolescents; dramatized videos combined with guided discussions enhance knowledge retention and may generate spillover effects among families and peers. However, to achieve sustainable impact, eSHEPP should be integrated into formal school curricula with active teacher facilitation and parental reinforcement. Policy and donor support will be critical to expand reach and address the growing burden of NCDs among youth in low- and middle-income countries.

## Supplementary Material

Supplementary Files

This is a list of supplementary files associated with this preprint. Click to download.

SupplementaryMaterial12and3.docx
SupplementaryMaterial4COREQchecklist.docx
SupplementaryMaterial5Codebook.docx
SupplementaryMaterial6Focusgroupdiscussionguide.docx

## Data Availability

The deidentified interview transcripts analyzed during the current study are not publicly available due to confidentiality concerns but may be made available from the corresponding author upon reasonable request and subject to approval from the ethics committee.

## Key messages regarding feasibility

### 1) What uncertainties existed regarding the feasibility?

Prior to this study, it was uncertain whether a multimedia, narrative-based digital health education program could be delivered in under-resourced public schools in Karachi without disrupting academic routines, using only basic equipment (laptop, speaker, projector) and an offline-capable mobile app. It was also unclear whether students would find the content engaging and acceptable.

### 2) What are the key feasibility findings?

- Students found eSHEPP highly acceptable, describing it as enjoyable, easy to understand, and a welcome break from traditional lectures (“*The atmosphere was very friendly*” – FGD2, Female).
- The program required minimal resources and fitted smoothly into school timetables (“*We don’t need expensive equipment or complicated setups, just basics like a laptop and speaker*” – FGD3, Male).
- Minor barriers included occasional technical difficulties (poor video/audio quality), background noise, and seating arrangements where older students blocked visibility for younger students.
- Students suggested expanding health topics, adding interactive app features (voice recording, games, playback controls), and slightly lengthening sessions for deeper discussion.

### 3) What are the implications of the feasibility findings for the design of the main study?

- A future definitive trial should standardise classroom logistics (seating arrangements, audio equipment) to address the identified barriers.
- Student preferences for weekly sessions and 7–10-minute videos should be tested; a trial arm with slightly longer sessions may be considered.
- Teacher involvement, valued for maintaining focus (especially in male schools), should be maintained but clearly distinguished from facilitator roles in trial protocols.
- Future research should assess feasibility in rural schools, private schools, and settings with lower digital connectivity, as this study was limited to urban public schools in Karachi.

## Abbreviations

COREQ: Consolidated Criteria for Reporting Qualitative Research
FGD: Focus Group Discussion
LMICs: Low- and Middle-Income Countries
mHealth: mobile health
NCDs: Noncommunicable Diseases
eSHEPP: School eHealth Education Program Pakistan
TTF: Task–Technology Fit
TAM: Technology Acceptance Model
WHO: World Health Organization
